# Targeting of Mutant Isocitrate Dehydrogenase in Glioma: A Systematic Review

**DOI:** 10.3390/cancers17162630

**Published:** 2025-08-12

**Authors:** Tyler A. Lanman, L. Nicolas Gonzalez Castro

**Affiliations:** 1 Center for Neuro-Oncology, Dana-Farber Cancer Institute, Boston, MA 02115, USA; 2 Department of Neurology, Harvard Medical School, Boston, MA 02115, USA; 3 Pappas Center for Neuro-Oncology, Massachusetts General Hospital, Boston, MA 02115, USA; 4 Department of Neurology, University of Washington, Seattle, WA 98195, USA; 5 Department of Neurology, Brigham and Women’s Hospital, Mass General Brigham, 60 Fenwood Rd, Boston, MA 02115, USA; 6 Center for Tumors of the Nervous System, Mass General Brigham Cancer Institute, Boston, MA 02114, USA

**Keywords:** glioma, IDH-mutant glioma, isocitrate dehydrogenase, IDH inhibitor, vorasidenib, ivosidenib, INDIGO, primary brain tumors, precision oncology, systematic review

## Abstract

**Background/Objectives**: Mutant isocitrate dehydrogenase (IDH) inhibitors represent a major advance in precision oncology. The recent Food and Drug Administration approval of vorasidenib for IDH-mutant glioma highlights its therapeutic potential in this setting. As this and other mutant IDH inhibitors enter the clinical setting, providers are tasked with staying informed of the evolving therapeutic landscape as more is learned about this unique class of medications. We aimed to summarize insights from preclinical studies and clinical trials exploring their use in IDH-mutant glioma. **Methods**: We reviewed notable preclinical studies establishing the rationale for targeting mutant IDH. We performed a systematic review of clincaltrials.gov to identify both completed and ongoing interventional IDH-directed trials in patients with IDH-mutant glioma. **Results**: We identified 8 published and 15 ongoing clinical trials evaluating IDH-directed therapies. IDH inhibitors have been shown to slow and, in some cases, reverse glioma tumor growth, with activity that may extend beyond their currently approved indications. The presence of contrast enhancement is consistently a negative predictor of response for ivosidenib and vorasidenib, although safusidenib and olutasidenib preliminarily may retain efficacy in these cases. Novel approaches such as IDH-directed vaccines and combination therapy using mutant IDH inhibitors with immunotherapy are currently under active investigation. **Conclusions**: Mutant IDH inhibition is a promising, well-tolerated, and evolving approach for many patients with IDH-mutant glioma. Ongoing research will clarify its optimal clinical utility and potentially expand its indication.

## Simple Summary

Mutant isocitrate dehydrogenase (IDH) inhibitors have introduced a new era in the field of IDH-mutant glioma. This systematic review identifies and summarizes key published and ongoing preclinical studies and clinical trials investigating the use of IDH-targeted therapies for these tumors. While mutant IDH inhibition represents a promising and well-tolerated therapeutic approach, its optimal clinical application is still being explored, with the potential for expanded indications as evidence evolves.

## 1. Introduction

Isocitrate dehydrogenase (IDH) mutations define a unique class of diffuse infiltrating gliomas affecting young and middle-aged adults. These gliomas are often diagnosed as low-grade tumors that, over years to decades, invariably progress to higher grade neoplasms with aggressive behavior and an expected fatal outcome [1]. The initial oncogenesis of IDH-mutant gliomas arises from aberrant oncogene expression in a setting of epigenetic dysregulation resulting from the inhibitory activity of D-2-hydroxyglutarate (D-2HG) levels, the oncometabolite produced by mutant IDH, on DNA and histone demethylases [2,3,4]. Thus, the reduction of D-2HG levels via small molecule inhibition of mutant IDH activity has been pursued as a therapeutic strategy in IDH-mutant glioma over the last decade, resulting in the FDA approval of the dual mutant-IDH1/2 inhibitor vorasidenib for the treatment of low-grade IDH-mutant glioma.

Here, we systematically review the available clinical evidence for the use of IDH inhibition for the treatment of newly diagnosed and recurrent IDH-mutant gliomas of all grades and histologies. We discuss preclinical breakthroughs that have led up to our current understanding of IDH in the pathogenesis of IDH-mutant glioma, progress in the development of IDH inhibitors, and studies evaluating the use, safety, and efficacy of various IDH inhibitors. Finally, we review novel approaches to IDH inhibition in combinatorial therapy, including IDH-targeted vaccine strategies, and summarize promising ongoing and planned IDH inhibition studies. Especially since the field of IDH-mutant glioma has been rapidly advancing in recent years, we hope that our review will equip clinicians, scientists, and patients with comprehensive, up-to-date knowledge of the past, present, and future of IDH-mutant targeted strategies for this unique tumor type.

### 1.1. Discovery of IDH Mutations and Their Role in Gliomagenesis

**Figure 1:**
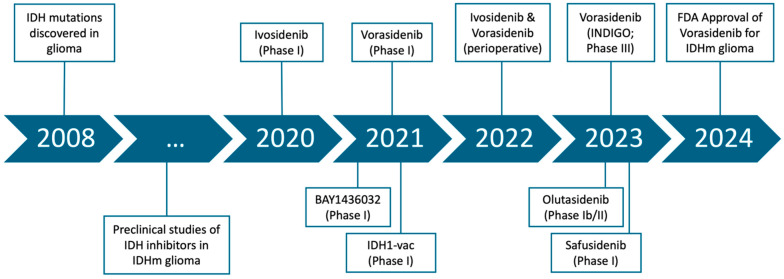
Development timeline for IDH inhibitors.

Our current understanding of IDH mutations and their role in tumorigenesis derives from extensive research spanning decades (Figure 1). As the different enzymatic reactions of the tricarboxylic acid cycle were first being characterized, scientists discovered in 1937 that isocitrate is converted to alpha-ketoglutarate, a reaction mediated by IDH [5]. IDH was subsequently found to have 3 isoforms: NADP+-dependent IDH1 (located in the cytosol and peroxisomes), NADP+-dependent IDH2 (in mitochondria), and NAD-dependent IDH3 (in mitochondria) [6], the first two catalyzing reversible reactions while the latter catalyzing an irreversible reaction. These enzymes were thought to be strictly associated with cellular metabolism until 2008, when genetic profiling of glioblastoma samples revealed the presence of IDH1 mutations in 12% of the analyzed samples, which originated from younger patients [7]. Subsequently, IDH mutations have been implicated in a variety of other tumor types, including sinonasal undifferentiated carcinoma (occurring in 49–82% of cases), angioimmunoblastic T-cell lymphoma (32% of cases), acute myeloid leukemia (AML; in 20%), cholangiocarcinoma (~20%), and at lower frequencies in other rare tumor types [8,9]. Yan et al. found that among glioma samples, R132H was the most common mutation in IDH1 and that IDH2 was also mutated at a lower frequency in gliomas [10]. Furthermore, they found that patients with gliomas harboring IDH mutations had improved survival compared to those without, with median overall survival (OS) over twice as long in those with IDH mutations compared to those without within glioblastoma and within grade 3 astrocytomas [10]. This profound prognostic discrepancy led the World Health Organization in its 2016 Classification of CNS Tumors to subdivide diffuse gliomas into two distinct categories: those with IDH mutations (IDH-mutant) and those without (IDH wild-type or IDHwt) [11]. IDH-mutant glioma is further subdivided into oligodendroglioma (defined by co-deletion of 1p19q) and astrocytoma (intact 1p19q).

Beyond just prognostic significance, IDH mutations were found to be pathophysiologically prominent driver mutations in the early development of gliomas [12]. Whereas wild-type IDH converts isocitrate to alpha-ketoglutarate, the mutant form of the enzyme then converts alpha-ketoglutarate to D-2HG [13]. This D-2HG accumulates at very high intracellular concentrations and acts as an oncometabolite through several mechanisms. Investigators found that the IDH mutation itself was sufficient to induce the glioma CpG-island methylator phenotype (G-CIMP) characteristic of low-grade IDH-mutant glioma [14]. This appears to be facilitated by D-2HG’s competitive antagonism of TET2, the primary enzyme for demethylating DNA [15]. D-2HG also prevents histone demethylation by inhibiting KDM4C (an enzyme in the JmjC family) [16]. In summary, D-2HG prevents key demethylation reactions from occurring, resulting in genome-wide and histone-wide hypermethylation. This epigenetic dysregulation leads to silencing of tumor suppressor genes and activation of oncogenes [17]. Notably, D-2HG accumulation results in chromosomal topology disruption, which uncovers a constitutive enhancer of platelet-derived growth factor receptor alpha (PDGFRA), leading to increased expression [4].

### 1.2. Development of IDH Inhibitors

Since mutated IDH is found exclusively in IDH-mutant tumor cells and plays a central role in gliomagenesis, this mutated enzyme was hypothesized to be an ideal therapeutic target and led to the development of treatments blocking mutant IDH (Figure 1). In 2012, Agios Pharmaceuticals identified the first small molecule selective inhibitor of mutated IDH (in this case specific to the R132H IDH1 mutation) via high throughput screening, with subsequent demonstrations of delayed growth and increased differentiation of stem-like tumor IDH-mutant glioma cells [18,19]. This drug decreased D-2HG concentrations and reduced in vivo flank tumor growth rates [19]. However, this drug was found to have non-ideal pharmacokinetic (PK) properties including rapid metabolism/clearance, so it was not selected to proceed into clinical trials [20]. Instead, second-generation IDH-mutant inhibitors were designed to overcome these issues. AG-120 (ivosidenib, brand name Tibsovo), another selective IDH1-R132H inhibitor, was found to have robust D-2HG reduction and more favorable pharmacokinetics, although relevant to glioma, had a brain penetrance of only 4.1% (although it was thought to have greater brain penetrance in glioma patients who have a compromised blood–brain barrier) [21]. Whereas IDH1 mutations are more frequent than IDH2 mutations in glioma, the reverse is true for AML [22]. Therefore, AG-221 (enasidenib, brand name Idhifa) was developed by Celgene Corporation/Agios Pharmaceuticals as a selective inhibitor of mutant IDH2 enzymes [23]. However, since ivosidenib and enasidenib each only targeted a single mutated isoform and neither had particularly high brain penetrance, Agios then developed AG-881 (vorasidenib, brand name Voranigo), which could inhibit both mutant IDH1 and IDH2 isoforms, and was found to have brain penetrance superior to those of its predecessors, with a brain tumor to plasma ratio of 1.33 [24]. Other notable mutant IDH inhibitors include BAY-1436032 (developed by Bayer Pharmaceutical but not presently under clinical development) [25], FT-2102 (olutasidenib, developed by Rigel Pharmaceuticals and Forma Therapeutics) [26], IDH305 (developed by Novartis Oncology but not presently under clinical development) [27], and DS-1001 (AB-291 or safusidenib, in development by Daiichi Sankyo) [28]. These drugs are all selective for IDH1 mutations.

### 1.3. Initial Clinical Evaluation of IDH Inhibitors

As of April 2025, three mutant IDH inhibitors (enasidenib, ivosidenib, and olutasidenib) have been FDA-approved for non-glioma indications. Due to initial promising preclinical results, the first-in-human study of IDH inhibition began in 2013, treating patients with AML and IDH2 mutations with the mutant IDH2 inhibitor, enasidenib [29]. This single-arm phase I/II trial demonstrated good tolerance of enasidenib and an objective response rate (ORR) of 40%, leading to the first mutant IDH inhibitor FDA approval in 2017 [30]. The mutant IDH1 inhibitor ivosidenib was simultaneously evaluated in patients with AML in a single-arm phase I study [31], a randomized double-blind phase III study in patients with cholangiocarcinoma [32], and a single-arm phase I trial in patients with myelodysplastic syndrome [33], leading to FDA approvals for these indications in 2018, 2021, and 2023, respectively. Finally, olutasidenib was approved for AML in 2022 based on the results of a phase I/II single-arm study that demonstrated similar response rate but longer response duration compared to previously reported ivosidenib outcomes [34].

## 2. Methods

**Figure 2:**
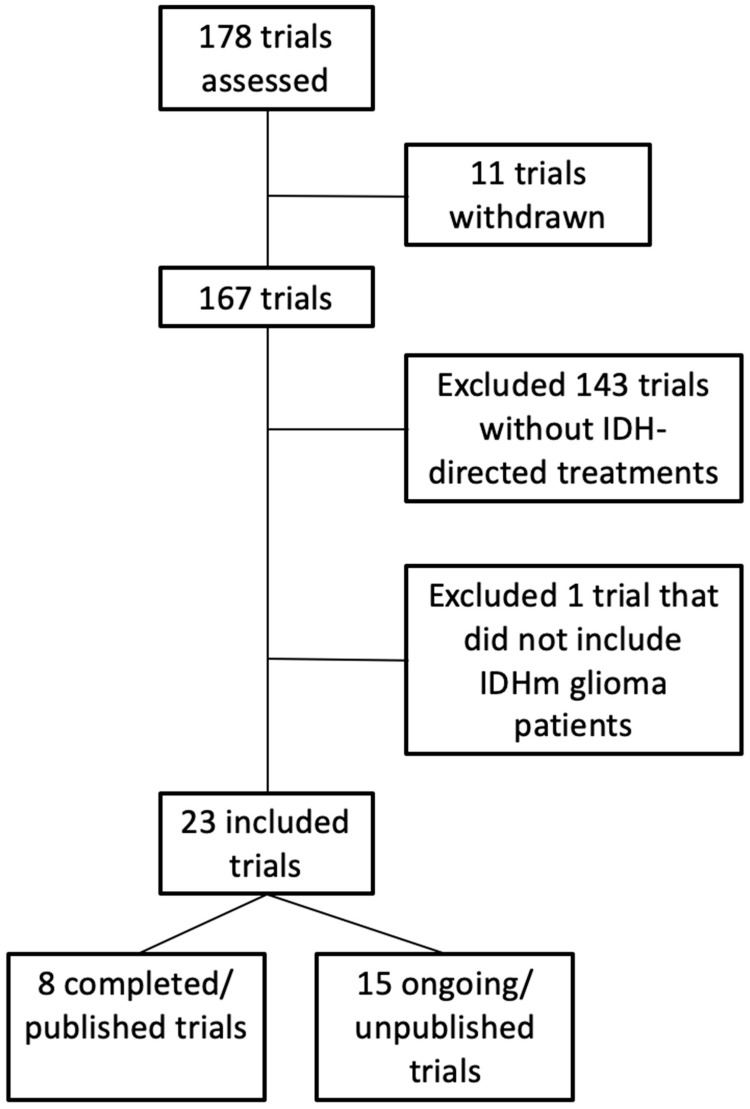
Consort diagram for systematic review.

A systematic review of registered trials (past, present, and future) was performed on https://clinicaltrials.gov/ on 28 June 2025, with the search criteria: “glioma AND (IDH OR IDH1 OR IDH2) AND AREA[StudyType](INTERVENTIONAL).” This query resulted in 178 studies, which were then screened for those that have not been canceled and those that included patients with glioma and had an IDH-directed therapy as part of the intervention (Figure 2). A total of 8 trials with published results (Table 1) and 15 trials that are ongoing/unpublished (Table 2) were identified. The following sections reflect trials from this query.

## 3. Results

### 3.1. Evaluation of IDH Inhibitors in Glioma

#### 3.1.1. Ivosidenib in IDH1-Mutant Advanced Glioma

Starting in 2014 as part of a larger trial that recruited patients with IDH mutations in any solid tumor type, this was the first IDH inhibitor trial to enroll patients with IDH-mutant glioma [35]. A total of 66 patients with an established diagnosis of IDH1 mutant glioma (grades 2–4) and progressive disease were enrolled. The primary objectives were to assess safety/tolerability and determine an optimal dose. In the dose escalation phase, the maximum tolerated dose was not reached, so 500 mg was selected for the expansion cohort based on pharmacokinetic and pharmacodynamic (PD) data. Since the presence of contrast enhancement on MRI is suggestive of higher grade, the expansion phase of this study separated patients into two cohorts: one with contrast-enhancing tumors and one without. Ivosidenib was generally well-tolerated. The most common adverse events (AE) were headache, fatigue, nausea/vomiting, seizure, and diarrhea. Grade 3–4 toxicities occurred in 19.7% of patients. Treatment-related AEs occurred in 59.1%, although most were grade 1–2. At the time of analysis, the progression-free survival (PFS) was 13.6 months for the non-enhancing cohort and 1.4 months for the enhancing cohort. In patients with IDH-mutant glioma, where disease stability is the primary goal and tumor shrinkage is not typically expected with treatment, disease control rate (DCR; best response rate of stable disease or better) is a reasonable outcome measure in place of overall response rates (ORR). This study reported 85.7% DCR in those with non-enhancing disease compared to 45.2% in those in the enhancing cohort. As brain tumor volumetric growth rate is an emerging potential radiographic correlate of disease progression [36,37], volumetric analysis was included as an exploratory endpoint for non-enhancing tumors. Here, tumor growth rate was reduced from 26 to 9% after initiating ivosidenib, with many patients experiencing either a growth plateau or reduction.

As the first published study of IDH inhibitor use in IDH-mutant glioma, this trial was pivotal for the field [35]. It demonstrated that ivosidenib 500 mg once daily was generally well-tolerated, associated with relatively high DCR, and led to a reduced tumor growth rate. It also notably suggested that those with enhancing tumors do not respond nearly as well as those with non-enhancing tumors.

#### 3.1.2. Safety and Therapeutic Activity of BAY1436032 in Patients with IDH1-Mutant Solid Tumors

This international single-arm dose escalation/expansion phase I trial enrolled 81 patients with IDH1-mutated solid tumors between 2016 and 2018. In the dose expansion phase (using 1500 mg and twice daily dosing), 25 were considered “low-grade glioma” (grade 2–3), and 13 were considered IDH-mutant “glioblastoma” (now classified as astrocytoma, IDH-mutant, grade 4) [38]. Regarding safety, 72% of patients experienced grade 3–4 adverse events (elevated liver enzymes, elevated lipase, headache, nausea, and diarrhea), resulting in dose modification in 31% of patients and discontinuation in 24%. The DCR was 29% and 54%, and ORR was 0% and 11% in the GBM and lower-grade cohorts, respectively. On review of the results from both the glioma and non-glioma cohorts, it was determined that the compound exhibited relatively low overall response rates and inadequate D-2HG reduction, resulting in the decision not to further develop BAY1436032 [39].

#### 3.1.3. Vorasidenib, a Dual Inhibitor of Mutant IDH1/2, in Recurrent or Progressive Glioma

As the first clinical trial investigating vorasidenib in patients with IDH-mutant glioma, this study began recruiting patients shortly after the activation of the ivosidenib trial [35]. This was similarly designed as a phase I single-arm study with dose escalation [40]. It recruited 52 adult patients with recurrent or refractory grade 2–4 IDH1- or IDH2-mutant glioma (22 non-enhancing and 30 enhancing), from 2015 to 2017. Due to dose-dependent toxicities, 300 mg was the maximum recommended dose for further investigation. Grade 3–4 toxicities were seen in 19.2% of patients, with the most common AEs overall being headache, elevated liver function tests, fatigue, nausea, and seizure. Treatment discontinuation and reduction occurred in 3.8% and 13.5% of patients, respectively. PFS was 36.8 and 3.6 months for those in the non-enhancing and enhancing cohorts, respectively. DCR was 90.9% and 58.6%, and ORR was 18.2% and 0% in non-enhancing and enhancing cohorts, respectively. Volumetric growth rate analysis was not reported. Although three patients with IDH2 mutations were recruited, the individual outcomes for these patients were not reported.

This trial confirmed the feasibility of vorasidenib for IDH-mutant glioma and demonstrated a longer PFS compared to the ivosidenib trial, although different patient populations and study designs prevent direct comparison [35]. It also provided further evidence that patients with contrast-enhancing disease do not appear to respond to IDH inhibition.

#### 3.1.4. A Vaccine Targeting Mutant IDH1 in Newly Diagnosed Glioma

A peptide vaccine directed against IDH1 R132H was developed and tested in a Phase 1 trial, where it was given to patients with grade 3–4 astrocytoma either after radiotherapy or in conjunction with adjuvant temozolomide [41]. This vaccine induced an immune response in 93% of patients and met its safety endpoint (all vaccine-related adverse events were limited to grade 1).The three-year progression-free rate was 63% and a relatively high rate of pseudoprogression (38%) was observed. This first-in-class trial provided the mechanistic basis for further IDH-targeted vaccine strategies.

#### 3.1.5. Vorasidenib and Ivosidenib Perioperative Phase 1 Trial

As the results from the ivosidenib and vorasidenib phase I studies were being analyzed, the tolerability and brain penetration of these compounds were being considered to select a candidate for a phase 3 trial [42]. Both appeared to be relatively well-tolerated and showed indications of efficacy in IDH-mutant glioma. Ivosidenib had a longer track record in oncology and was already FDA-approved in 2018 for AML. On the other hand, vorasidenib had potentially better brain penetrance and could inhibit both IDH1 and IDH2 mutant isoforms rather than just IDH1. Direct comparison between the two drugs was impossible due to differences in trial designs and patient populations. To help resolve this issue, a phase 1 perioperative trial was designed as a head-to-head comparison, evaluating PK, PD, and safety. The primary endpoint was D-2HG reduction in resected tumors. Patients with a known diagnosis of IDH1-mutant glioma who required surgery for tumor recurrence were randomized to receive vorasidenib (at a dose of either 10 or 50 mg daily), ivosidenib (either 250 mg twice daily or 500 mg daily), or no treatment prior to surgery. Patients with enhancing tumors were excluded due to lack of demonstrable efficacy in the earlier phase 1 studies. Following surgery, patients would subsequently be continued on vorasidenib or ivosidenib until recurrence. In total, 24 patients received vorasidenib, and 25 received ivosidenib.

Although the tumor:plasma ratios were higher for vorasidenib (1.57 for vorasidenib 50 mg vs. 0.089 for ivosidenib 500 mg daily), ivosidenib actually reached a higher tumor drug concentration given its higher dose (233 ng/g for ivosidenib vs. 110 ng/g for vorasidenib). Similarly, robust D-2HG reduction was seen in both drugs, with 92.6% reduction with vorasidenib 50 mg daily and 91.1% reduction with ivosidenib 500 mg daily. Vorasidenib 50 mg had a DCR and ORR of 85.7% and 42.9%, respectively, while ivosidenib 500 mg had DCR and ORR of 100% and 35.7%. Exploratory analyses revealed an association between radiographic tumor response and elevated DNA 5hmC content, highlighting the role of TET enzymes in IDH-mutant gliomagenesis and suggesting the potential use of demethylating agents in IDH-mutant glioma. They also found that D-2HG reduction via IDH inhibitors resulted in the upregulation of antitumor immune genes, suggesting that IDH inhibitors might increase immune activation in IDH-mutant glioma [43]. This finding invoked the possibility of combining IDH inhibition with immune-directed therapies.

Ultimately, this study’s objective was to provide a multimodal comparison of ivosidenib and vorasidenib, determining which one would move forward into a larger phase 3 trial. Although ivosidenib showed similar results to vorasidenib, the latter was chosen because it 1) had broader isoform coverage (it inhibits both mutant IDH1 and IDH2), 2) had slightly better and more consistent D-2HG inhibition, and 3) had nominally higher ORR.

#### 3.1.6. Vorasidenib in IDH1- or IDH2-Mutant Low-Grade Glioma

After three phase 1 trials of IDH inhibitors in IDH-mutant glioma and a perioperative head-to-head comparison, a double-blind, placebo-controlled phase 3 trial was designed to assess efficacy of vorasidenib in low-grade tumors. This trial was called INDIGO (“INvestigating VorasiDenib In GliOma”) [44]. A total of 331 patients were recruited between 2020 and 2022, with 168 patients randomized to receive vorasidenib 40 mg daily (a slightly lower dose than the 50 mg dose in previous trials) and 163 were randomized to placebo. Inclusion criteria were: patients with residual or recurrent IDH-mutant glioma, at least 12 years old, Karnofsky Performance Scale (KPS) of at least 80, were between 1–5 years out from surgery, were deemed to be a candidate for a watch-and-wait approach, and had measurable non-enhancing (any enhancement had to be nonmeasurable) disease. PFS was the primary endpoint for this trial. Secondary endpoints included time to next intervention (TTNI), safety, volumetric growth rates, quality of life, seizure control, and neurocognition. No deaths had occurred in either group by the time of publication, so overall survival comparisons were not possible.

With a median follow-up time of 14 months overall, vorasidenib showed a median PFS of 27.7 months compared to 11.1 months with placebo, thereby achieving the primary endpoint. TTNI was not reached in the vorasidenib arm, and was 17.8 months in the placebo arm, demonstrating that vorasidenib could effectively delay the use of more toxic treatment modalities, such as radiotherapy and chemotherapy. Vorasidenib was overall well-tolerated. Grade 3–4 adverse events (predominantly elevated liver enzymes) occurred in 22.8% of patients in the vorasidenib compared to 13.5% in the placebo arm. Dose reduction and discontinuation occurred in 10.8% and 3.6% of patients in the vorasidenib arm, respectively.

This was a paradigm-shifting study for the field of IDH-mutant glioma, and it facilitated FDA approval for vorasidenib in IDH-mutant glioma on August 6, 2024. Specifically, the label defined its indication for patients with IDH1- or IDH2-mutant astrocytoma or oligodendroglioma following surgery [45]. This approval represented the first approved systemic treatment for low-grade glioma in over 20 years and was the first approved molecularly targeted therapy for this tumor type.

#### 3.1.7. Phase I Study of IDH1 Inhibitor DS-1001 in Recurrent or Progressive IDH1-Mutant Glioma

This Japanese study, published in 2023, evaluated DS-1001 (safusidenib), a selective inhibitor of mutant IDH1 [46]. Enrolling 47 patients, they found that DS-1001 was relatively well-tolerated, with grade 3 AEs occurring in 42.6% of patients (predominantly skin hyperpigmentation, diarrhea, pruritus, alopecia, arthralgia, nausea, headache, rash, and dry skin). Non-enhancing tumors had a 33.3% ORR, but importantly, they found that 17.1% of those with enhancing tumors had an objective response. Specifically, two out of 35 patients with enhancing disease had CR, and four achieved PR.

Prior trials with ivosidenib and vorasidenib showed disappointing efficacy in patients with enhancing tumors. It had, therefore, been hypothesized that enhancement is a marker of more aggressive disease, and by extrapolation that the tumor had likely developed growth mechanisms that were distinct from those enabled by the IDH mutation, making the latter a less attractive therapeutic target in high-grade tumors. However, the results from the safusidenib trial appear to contradict that hypothesis. Since the adverse event profile and efficacy in enhancing tumors were discrepant from vorasidenib and ivosidenib, this provided evidence that different IDH inhibitors may have different therapeutic spectrums [47].

#### 3.1.8. Olutasidenib (FT-2102) in Relapsed or Refractory IDH1-Mutant Glioma

As part of a larger international basket trial investigating olutasidenib in solid tumors, this phase 1b/2 study reported outcomes in 26 patients with IDH1-mutant glioma [48]. Notably, this was a heavily pre-treated population, and 88% of the patients had enhancing disease. With 150 mg twice daily as the chosen dose for the phase 2 portion, this trial did not meet the primary endpoint of ORR (8% overall) although the DCR was 48%. Similar to other trials of IDH-mutant inhibitors, olutasidenib was well-tolerated, with grade 3–4 adverse events (mostly elevated liver enzymes) occurring in 41% of patients. Dose reductions occurred in 15% of patients, interruptions occurred in 19%, and there were no discontinuations due to adverse events.

### 3.2. Retrospective Studies

The FDA approval of vorasidenib in 2024 ushered in new treatment paradigms for IDH-mutant glioma. While their safety and efficacy seemed promising in clinical trials leading up to the approval, it remained unclear how IDH inhibitors would perform in the real world. Each clinical trial had specific inclusion and exclusion criteria, which allowed for more homogenous populations but also limited their external validity. Following approval, several reviews and editorials weighed the merits or risks of extrapolating from the trials to specific clinical situations not included in the trials [49,50,51,52,53,54]. The relevance of certain clinical and demographic features such as grade (since the INDIGO study only allowed grade 2 tumors), extent of disease/extent of resection (since INDIGO required measurable disease), presence of contrast enhancement (since phase 1 trials showed limited efficacy of ivosidenib/vorasidenib in contrast-enhancing tumors), and efficacy after other treatments (since INDIGO required prior surgery but excluded those with other prior treatments such as chemotherapy and radiotherapy) was debated.

There was a period when IDH inhibition seemed promising based on the initial phase 1 studies but vorasidenib had not yet been approved. Until that point, providers were only able to prescribe off-label ivosidenib to their patients with IDH-mutant glioma. To provide more insight into real-world IDH inhibitor use, several retrospective studies were published, reporting institutional experience using off-label ivosidenib outside the strict confines of clinical trial design. Peters et al. reported the Duke University experience with ivosidenib in 30 patients with IDH-mutant glioma [55]. In this cohort, including patients with WHO grade 2–4 tumors, ivosidenib was well-tolerated, had a DCR of 90.9%, and those without enhancing disease had better responses. Kamson et al. reported the Johns Hopkins experience using ivosidenib in 12 patients with WHO grade 2–3 tumors [56]. Here, they performed a detailed volumetric analysis of MRI exams, finding that tumor growth rate decreased from 8.0cc/year prior to ivosidenib to −1.2cc/year during ivosidenib use. They found that the median time to best response was 11.2 months, suggesting that for clinically stable patients on ivosidenib, a period of 6–12 months should be permitted to fully assess response. We reported our experience with ivosidenib in 74 patients with tumors spanning grades 2, 3, and 4 at Mass General Brigham and the Dana-Farber Cancer Institute [57]. Here, we found that ivosidenib was well-tolerated and had a DCR of 77%. Subset analyses showed that those without contrast enhancement had significantly better responses to ivosidenib, and initial-line ivosidenib use (compared to in the recurrent setting) had better response (although this might have been driven by the higher proportion of enhancing disease in subsequent-line use; 45% compared to 12%). Patients with enhancing disease that was entirely resected by the time of ivosidenib initiation (*n* = 4) had DCRs similar to those who had never had enhancing disease. We notably did not detect significant differences in response to ivosidenib for lower grades (2 vs. 3), age (older vs. younger than 40 years old), histology (astrocytoma vs. oligodendroglioma), or IDH mutation (canonical vs. noncanonical). Although these three studies had the inherent limitation of retrospective design, they demonstrated the real-world safety and efficacy of ivosidenib in IDH-mutant glioma and deepened our understanding of which patients may or may not respond to IDH inhibitors.

### 3.3. Updates on Tumor Volumetrics and Seizure Control with IDHi

Since the initial publication of clinical trials investigating IDH inhibitors in IDH-mutant glioma, several notable updates have been presented. Volumetric analysis of 21 patients from the phase 1 ivosidenib study [35] demonstrated that compared to 2D tumor measurements, 3D analysis showed significantly longer PFS, slower tumor growth rate, and was more consistent (less “yo-yo-ing” of measurements over time) [58]. This suggested that moving forward, response assessment might benefit from transitioning from 2D to 3D measurements as a more reliable radiographic biomarker of disease. Updated results from the perioperative trial of ivosidenib and vorasidenib [42] were presented in 2024 and reported that ORR was slightly higher for vorasidenib than ivosidenib (46% compared to 27%), preliminarily suggesting that vorasidenib might be more efficacious than ivosidenib [59]. However, this has yet to be corroborated by larger studies. Secondary endpoint results for the INDIGO trial were presented in 2024 [60]. They reported that at a median follow-up of 14.2 months, quality of life (as measured by the FACT-Br questionnaire) was similar between arms (mean score 163.3 in the vorasidenib arm compared to 161.4 in the placebo arm), and neurocognition was similar between arms (with no evidence of treatment-related deterioration). Another updated INDIGO analysis also reported that vorasidenib had a significantly lower rate of on-treatment seizures per person-year (18.2 vs. 51.2%, *p* = 0.0263) [61], suggesting that either tumor growth in the placebo arm led to higher seizure rates and/or that IDH inhibition actually has some antiseizure properties, as originally predicted based on preclinical work [62]. Finally, updated primary efficacy results from INDIGO were presented, reporting that both PFS and TTNI remained significantly in favor of the vorasidenib arm [61]. In 2022, Servier opened an Expanded Access Program (EAP) to provide vorasidenib to patients with IDH-mutant glioma outside of the INDIGO trial. Results from 71 patients who started vorasidenib under this program were presented in 2024 [63]. They reported that 87% of patients remained on vorasidenib at the time of analysis; 9.5% discontinued for radiographic or clinical progression, and no patients discontinued for treatment-related toxicity.

## 4. Discussion

### Future Directions and Ongoing Studies

Since the initial IDH inhibitor trials leading to the FDA approval of vorasidenib were published, a plethora of subsequent trials have either been planned or are already underway. These trials hope to expand indications for IDH inhibitors, investigate new IDH inhibitors, and evaluate novel approaches to inhibiting mutant IDH. Clinicians’ familiarity with these trials can facilitate access for patients to new investigational therapeutic avenues before they are widely available and provide an overview of the most salient unanswered questions in the field that will hopefully be elucidated.

As of 8 June 2025, two mutant IDH-directed trials are not yet recruiting, five are currently recruiting (three of which are available in the United States), five are “active, not recruiting” (indicating that maturation of outcome data and/or analysis are pending), and three are indicated as completed but published results are not yet available. Some of these trials explore alternative methods and settings to utilize vorasidenib beyond its current indications, such as combining it with chemotherapy (NCT06478212) or immunotherapy (NCT05484622), and include patients with grade 4 and/or enhancing tumors. Although many immune-directed treatments have largely been unsuccessful in glioma, recent work has demonstrated that the IDH mutation leads to immunosuppression in the tumor microenvironment [64,65,66] and that IDH inhibitors can reverse this immunosuppressive environment [67]. Perhaps IDH inhibition will facilitate the long-awaited breakthrough for immunotherapy in glioma, but further investigation is necessary. Some hope to provide further evidence for alternative (perhaps safer or more efficacious) IDH inhibitors such as olutasidenib (NCT06161974), safusidenib (NCT05577416), and DS-1001b (NCT04458272). Some specifically include pediatric and adolescent patients to explore IDH inhibition in a younger population than initially assessed (NCT06780930, NCT06478212, NCT06161974, NCT04195555). In addition to small-molecule inhibitors of mutant IDH, peptide vaccines have been developed to induce a T-cell response against cells with IDH mutations [68,69]. The NOA16 trial evaluated a peptide vaccine in 33 patients with newly diagnosed WHO grade 3–4 IDH-mutant astrocytoma [41]. It met its primary safety endpoint (vaccine-related adverse events were limited to grade 1), induced immune responses in 93% of patients, and had 3-year progression-free and death-free rates of 0.63 and 0.84, respectively. Several other ongoing or future trials using IDH-directed peptide vaccines in IDH glioma (NCT05609994, NCT03893903, NCT02193347) are expected to provide a better understanding of this novel and promising approach.

## 5. Conclusions

The discovery of IDH mutations and their oncogenic role in glioma has revolutionized the diagnostic, prognostic, and therapeutic landscape of these unique tumors. Preclinical breakthroughs have paved the way for the development of a myriad of IDH-targeted therapies, including small molecule IDH inhibitors (directed against IDH1 and IDH2 mutations) and vaccines that elicit immunogenic responses against glioma cells with these mutations. The landmark INDIGO trial led to the first FDA approval of the IDH1/2-mutant inhibitor, vorasidenib, for IDH-mutant glioma [44], representing the first approval of a systemic targeted therapy for this tumor type. With this development, the neuro-oncology community is now trying to ascertain the full therapeutic spectrum of IDH-mutant targeted interventions. Questions facing the field relate to how tumor grade, age, enhancement, and extent of resection should be factored into clinical decisions in the era of IDH inhibitors [53,57]. In addition to questions related to treatment efficacy, there are areas of uncertainty in regard to IDH inhibitors and their impact on patient quality of life. Our group recently reviewed the current understanding regarding the fertility/reproductive implications of IDH inhibitors, stressing the need for clinicians to adequately discuss these potential reproductive implications and document conversations when considering initiating an IDH inhibitor [70]. Other articles also provide recommendations for practical management strategies and advice using IDH inhibitors [71,72].

In the quest to provide well-tolerated and effective treatment options for IDH-mutant glioma, recent advances provide adequate reasons to be optimistic. Although additional follow-up time is needed to ascertain overall survival benefit and further studies are warranted to optimize the use of IDH-mutant targeting therapies, the road forward is promising.

## Figures and Tables

**Table 1:** Published IDH inhibitor studies.

Treatment & Date Published	NCT Identifier	Study Design	Population	Sample Size and Timeline	Results	Safety *	Impact
Ivosidenib (2020)	NCT02073994	− Phase 1 − Single arm − Dose escalation & expansion	G2–4 IDHm glioma, including NE and E tumors	*n* = 66 Median treatment duration: 18.4 mo (NE) and 1.9 mo (E)	NE: PFS 13.6 mo, DCR 91%, ORR 3% E: PFS 1.4 mo, DCR 45%, ORR 0%	Grade ≥ 3 TEAE: 19.7% Headache, fatigue, nausea	− First to demonstrate feasibility/tolerability of an IDHi in IDHm glioma − NE tumors seemed to respond better than E
BAY1436032 (2021)	NCT02746081	− Phase 1 − Single arm − Dose escalation & expansion	G2–4 IDHm glioma, some NE but predominantly E tumors	*n* = 49 Median treatment duration: 5.5 mo for those with SD or better	G2–3: 3 mo PFS 31%, DCR 54%, ORR 11% G4: 3 mo PFS 22%, DCR 29%, ORR 0%	Grade ≥ 3 TEAE: 72% Headache, diarrhea, nausea	− First clinical study of this drug in glioma − Not being developed further based on results
Vorasidenib (2021)	NCT02481154	− Phase 1 − Single arm − Dose escalation & expansion	G2–4 IDHm glioma, including NE and E tumors	*n* = 52 Median treatment duration: 26.8 mo (NE) and 3.3 (E)	NE: PFS 36.8 mo, DCR 91%, ORR 18.2% E: PFS 3.6 mo, DCR 57%, ORR 0%	Grade ≥ 3 TEAE: 19.2% Headache, transaminitis, fatigue	− First clinical study of this drug in glioma − Reiterated lack of response in E tumors
IDH1-vac (2021)	NCT02454634	− Phase 1 − 3 arms (using different combinations of XRT and TMZ) − Single dose	G3–4 IDHm astrocytoma including NE and E tumors	*n* = 33 94% of patients reached end of treatment Median follow-up time: 46.9 mo	3 year progression-free rate: 63% 3 year death-free rate: 0.84%	Grade ≥ 3 TEAE: 0% Injection site reactions, skin/subcutaneous tissue disorders	− First-in-human study using an IDH-targeted vaccine in IDHm glioma − Had promising safety and immunogenic response rates but survival/efficacy to be determined
Vorasidenib vs. Ivosidenib (2023)	NCT03343197	− Phase 1 − 3 arms (vora, ivo, none) − Perioperative	NE G2–3 IDHm glioma (excluded E tumors)	*n* = 24 (vora); *n* = 25 (ivo) Median treatment duration: 14.3 mo (vora), 15.1 mo (ivo)	Tumor [2-HG] reduced by 93% (vora) and 91% (ivo) Vora: DCR 88%, ORR 29% Ivo: DCR 91%, ORR 27.3%	Grade ≥ 3 TEAE: 29.2% (vora) and 24% (ivo) Nausea, headache, diarrhea	− Although vora and ivo had comparable [2-HG] reduction and response rates, vora was selected to move forward for Phase 3 study
Vorasidenib (2023)	NCT04164901	− Phase 3 − 2 arms (vora, placebo) − Randomized, double-blind	NE G2 IDHm glioma (excluded E tumors)	*n* = 168 (vora); *n* = 163 (placebo) Median follow-up: 14.2 mo	Vora: PFS 27.7 mo, DCR 94%, ORR 10.7 Placebo: PFS 11.1 mo, DCR 91%, ORR 2.5%	Grade ≥ 3 TEAE: 22.8% Transaminitis, COVID-19, fatigue	− In this large study, vora improved PFS and TTNI, and was relatively tolerable − This study led to FDA approval of vora for IDHm glioma
Safusidenib (2023)	NCT05577416	− Phase 1 − Single arm − Dose escalation	G2–4 IDHm glioma, including NE and E tumors	*n* = 47 Median treatment duration: 91.2 mo (NE) and 7.3 mo (E)	NE: PFS not reached; DCR 100%, ORR 33.3% E: PFS 10.4w, DCR 51%, ORR 17.1%	Grade ≥ 3 TEAE: 42.6% (all grade 3, none higher) Skin hyperpigmentation, pruritis, alopecia	− First clinical study of this drug in glioma − First trial to show preliminary efficacy of IDHi in E IDHm glioma
Olutasidenib (2023)	NCT03684811	− Phase 1b/2 − Single arm − Single dose	G2–4 IDHm glioma, some NE but predominantly E tumors	*n* = 26 Median treatment duration: 4.2 mo	PFS 1.9 mo, DCR 48%, ORR 8%	Grade ≥ 3 TEAE: 42% Nausea, fatigue, transaminitis	− First clinical study of this drug in glioma − Preliminary suggestion of efficacy in E glioma

DCR = disease control rate (stable disease + minor response + partial response + complete response); E = enhancing; IDHm = IDH-mutant; ivo = ivosidenib; NE = nonenhancing; ORR = objective response rate (complete response + partial response + minor response); PFS = progression-free survival; SD = stable disease; TEAE = treatment-emergent adverse event.; TTNI = time to next intervention; vora = vorasidenib. * This column includes rates of Grade ≥ 3 toxicities and lists the 3 most common toxicities for each trial.

**Table 2:** IDH inhibitor studies in development.

Trial Name	NCT Identifier	Location	Study Design	Status *	Population	Objectives/Impact
Vorasidenib Maintenance for IDH Mutant Astrocytoma	NCT06809322	Europe	− Phase 3 − 2 arms (vorasidenib, placebo) − Randomized, blinded	Not yet recruiting	− ≥18 years − G2–3 IDHm astrocytoma − E or NE − Upfront	Assesses whether vorasidenib improves PFS immediately following first-line radiotherapy and chemotherapy (regimen determined by treating physicians)
ViCToRy: Vorasidenib in Combination With Tumor Specific Peptide Vaccine for Recurrent IDH1 Mutant Lower Grade Gliomas	NCT05609994	Single institution (Duke)	− Phase 1 − Single arm (vorasidenib combined with peptide vaccine) − Open label, non-randomized	Not yet recruiting	− ≥18 years − G2–3 IDH1R132H-mutant glioma − NE − First recurrence	Assesses safety and efficacy of a novel IDH-targeted peptide vaccine in combination with an IDHi
Vorasidenib Maintenance for IDH Mutant Astrocytoma	NCT06780930	China, Taiwan	− Phase 3 − 2 arms (vorasidenib, placebo) − Randomized, blinded	Recruiting	− ≥12 years − G2 IDHm glioma − NE − Between 1–5y from surgery	Verifies results of INDIGO, but in a Chinese/Taiwanese population
Vorasidenib in Combination With Temozolomide (TMZ) in IDH-mutant Glioma	NCT06478212	International (including US)	− Phase 1b/2 − Dose escalation & expansion arms − Open label, non-randomized	Recruiting	− ≥12 years − G2–4 IDHm glioma − E or NE − Upfront or recurrent	Assesses safety and efficacy of combining an IDHi with temozolomide chemotherapy after completion of radiation and/or chemotherapy or as therapy at first recurrence.
Study of Olutasidenib and Temozolomide in HGG	NCT06161974	International (including US)	− Phase 2 − Single arm with single dose − Open label, non-randomized	Recruiting	− ≥12 years − IDH1-mutant G3–4 astrocytoma, DIPG, and primary thalamic and spinal cord HGG − E or NE − Upfront	Assesses efficacy of combining this IDHi with chemotherapy after completion of radiotherapy in children and young adults
A Study of AB-218 (Safusidenib) in Patients With IDH1 Mutated Low Grade Glioma	NCT05577416	Single institution (Royal Melbourne Hospital in Australia)	− Phase 0/2 − Perioperative arm (Part A) and post-operative arm (Part B) − Open label, non-randomized	Recruiting	− ≥18 years − IDH1-mutant low-grade glioma − E or NE − Upfront	Assesses feasibility, PK and PD of safusidenib using a perioperative design
Study of Vorasidenib and Pembrolizumab Combination in Recurrent or Progressive IDH-1 Mutant Glioma	NCT05484622	US	− Phase 1 − 3 open-label arms (vorasidenib, vorasidenib + pembrolizumab, or neither) − Safety lead-in and randomized perioperative phases	Recruiting	− ≥18 years − G2–3 IDH1R132H-mutant glioma − E or NE − Recurrent/progressive	Ascertains recommended combination dose of an IDHi in combination with immunotherapy and then assesses safety and feasibility of this combination in those with enhancing disease
Safusidenib Phase 2 Study in IDH1 Mutant Glioma	NCT05303519	US	− Phase 2 − Multiple arms for different doses of safusidenib − Open-label, randomized	Active, not recruiting	− ≥18 years − G2–3 IDH1-mutant (IDH1 R132H or R132C) glioma − E or NE − Recurrent/progressive	Assesses optimal dose for safusidenib and then assesses efficacy, safety, and PK of this drug
A Study of HMPL-306 in Advanced Solid Tumors With IDH Mutations	NCT04762602	US, Spain	− Phase 1 − Dose escalation and expansion arms − Open label, non-randomized	Active, not recruiting	− ≥18 years − IDHm “low grade glioma” − E or NE − Recurrent/progressive	Assesses safety, tolerability, PK, PD, and preliminary efficacy of a novel IDHi that targets both mutant IDH1 and IDH2
Ivosidenib in Treating Patients With Advanced Solid Tumors, Lymphoma, or Histiocytic Disorders With IDH1 Mutations (A Pediatric MATCH Treatment Trial)	NCT04195555	International (including US)	− Phase 2 − Single arm − Open label, non-randomized	Active, not recruiting	− 12 months to 21 years -IDHm glioma − E or NE − Recurrent/progressive	Assesses long-term safety, tolerability, pharmacokinetics, and pharmacodynamics of ivosidenib in a pediatric/adolescent population
A Study of DS-1001b in Patients With Chemotherapy- and Radiotherapy-Naive IDH1 Mutated WHO Grade II Glioma	NCT04458272	Japan	− Phase 2 − Single arm − Open label, nonrandomized	Active, not recruiting	− ≥20 years − G2 IDH1R132H-mutated glioma − NE − No prior anticancer treatment	Assesses safety and efficacy of safusidenib in the upfront (nontreated) setting.
Study of LY3410738 Administered to Patients With Advanced Solid Tumors With IDH1 or IDH2 Mutations	NCT04521686	International (including US)	− Phase 1 − Multiple arms for different doses (although 2 cohorts will get drug in combination with gemcitabine/cisplatin or durvalumab) − Dose escalation and expansion − Open label	Active, not recruiting	− ≥18 years − G2 IDH1R132H-mutated glioma − E or NE − Upfront or recurrent/progressive	Assesses safety and efficacy of a mutant IDH1/2 inhibitor either as monotherapy or in combination with chemotherapy or immunotherapy
Ivosidenib (AG-120) With Nivolumab in IDH1 Mutant Tumors	NCT04056910	Single institution (UPMC Hillman Cancer Center in Pennsylvania)	− Phase 2 − Single arm (ivosidenib + nivolumab) − Open label, non-randomized	Completed, not yet published	− ≥18 years − Enhancing IDHm glioma − Recurrent/progressive	This will be the first trial evaluating the combination of IDH inhibition and immunotherapy
AMPLIFYing NEOepitope-specific VACcine Responses in Progressive Diffuse Glioma	NCT03893903	Germany	− Phase 1 − 3 open label arms (IDH1R132H peptide vaccine, avelumab, or both combined) − Open label, randomized	Completed, not yet published	− ≥18 years − G2-4 IDH1R132H-mutated astrocytoma (1p19q intact) − E or NE − Recurrent/progressive	Assesses safety and immunogenicity of a novel IDHm-directed peptide vaccine, either alone or in combination with immunotherapy
IDH1 Peptide Vaccine for Recurrent Grade II Glioma	NCT02193347	Single Institution (Duke)	− Phase 1 − Single arm (peptide vaccine + Tetanus + TMZ) − Open label, nonrandomized	Completed, not yet published	− ≥18 years − G2 IDH1R132H-mutated glioma − E or NE − Recurrent/progressive	Assesses safety and immunogenicity of a novel IDHm-directed peptide combined with TMZ +/− RT

E = enhancing; IDHm = IDH-mutant; NE = nonenhancing; PD = Pharmacodynamics; PK = Pharmacokinetics. * Status as of 5/8/25.
